# Application of a JEG-3 organoid model to study HLA-G function in the trophoblast

**DOI:** 10.3389/fimmu.2023.1130308

**Published:** 2023-03-15

**Authors:** Bai-Mei Zhuang, Dan-Dan Cao, Xiao-Feng Liu, Li Wang, Xiao-Li Lin, Yong-Gang Duan, Cheuk-Lun Lee, Philip C. N. Chiu, William S. B. Yeung, Yuan-Qing Yao

**Affiliations:** ^1^ Medical School of Chinese People’s Liberation Army, Chinese People’s Liberation Army General Hospital, Beijing, China; ^2^ Shenzhen Key Laboratory of Fertility Regulation, The University of Hong Kong-Shenzhen Hospital, Shenzhen, Shenzhen, Guangdong, China; ^3^ Department of Obstetrics and Gynecology, Affiliated Shenzhen Maternity and Child Healthcare Hospital, Southern Medical University, Shenzhen, China; ^4^ Department of Obstetrics and Gynecology, HKU Li Ka Shing (LKS) Faculty of Medicine, The University of Hong Kong, Hong Kong, Hong Kong SAR, China; ^5^ Department of Obstetrics and Gynecology, The First Medical Centre, Chinese People’s Liberation Army General Hospital, Beijing, China

**Keywords:** trophoblast organoid, human leucocyte antigen G, trophoblast differentiation, placentation, pregnancy

## Abstract

The human placenta is a unique temporary organ with a mysterious immune tolerance. The formation of trophoblast organoids has advanced the study of placental development. HLA-G is uniquely expressed in the extravillous trophoblast (EVT) and has been linked to placental disorders. With older experimental methodologies, the role of HLA-G in trophoblast function beyond immunomodulation is still contested, as is its role during trophoblast differentiation. Organoid models incorporating CRISPR/Cas9 technology were used to examine the role of HLA-G in trophoblast function and differentiation. JEG-3 trophoblast organoids (JEG-3-ORGs) were established that highly expressed trophoblast representative markers and had the capacity to differentiate into EVT. CRISPR/Cas9 based on HLA-G knockout (KO) significantly altered the trophoblast immunomodulatory effect on the cytotoxicity of natural killer cells, as well as the trophoblast regulatory effect on HUVEC angiogenesis, but had no effect on the proliferation and invasion of JEG-3 cells and the formation of TB-ORGs. RNA-sequencing analysis further demonstrated that JEG-3 KO cells followed similar biological pathways as their wild-type counterparts during the formation of TB-ORGs. In addition, neither HLA-G KO nor the exogenous addition of HLA-G protein during EVT differentiation from JEG-3-ORGs altered the temporal expression of the known EVT marker genes. Based on the JEG-3 KO (disruption of exons 2 and 3) cell line and the TB-ORGs model, it was determined that HLA-G has a negligible effect on trophoblast invasion and differentiation. Despite this, JEG-3-ORG remains a valuable model for studying trophoblast differentiation.

## 1 Introduction

The maternal-fetal interface has a complex microenvironment for semi-allogeneic crosstalk between mother and fetus during pregnancy (1). The human placenta is derived from the trophectoderm (TE) of the blastocyst, which is the predominant component of the interface. The trophectoderm generates the cytotrophoblast (CTB), which differentiates into the syncytiotrophoblast (ST) and the extravillous trophoblast (EVT) following embryo implantation (2). EVTs migrate and infiltrate the maternal decidua to anchor the placenta and remodel the spiral arteries (2). Unquestionably, placentation dysfunction is the leading cause of pregnancy disorders that may be fatal. Therefore, it is essential to understand the mechanisms during placental development.

Several *in vitro* models of placentation have been created over the past few decades (1). Long and widely employed to investigate trophoblast functions, such as invasion, two-dimensional (2D) culture of trophoblast cell lines was the method of choice for decades. However, the majority of these cell lines are derived from abnormal cells that cannot reflect the physiological, developmental, and biological characteristics of trophoblast *in vivo*. Okae et al., were the first to report the derivation of the widely accepted human trophoblast stem cells (TSCs) from blastocysts and first-trimester trophoblast digests in 2018 (3). More recently, three-dimensional (3D) organoid models were successfully created from primary placenta tissues that closely resembled normal first-trimester placentas (4, 5). CTs can proliferate indefinitely in the presence of activating (Wnt and epidermal growth factor) and inhibiting (Rho-associated protein kinases, histone deacetylase, and TGF-beta) signals; however, when switched to a differentiation medium supplemented with forskolin or NRG1, hCG^+^ multinucleated STs or HLA-G^+^ migratory EVTs can be generated, respectively. As the collection of primary placenta tissues is somewhat ethically restricted and scarce, Dietrich et al., demonstrated that JEG-3 choriocarcinoma cells can be propagated as a 3D organoid (6). Accordingly, they demonstrated that the JEG-3 organoid can be established and directed to undergo EVT differentiation, indicating its viability as a model for investigating the mechanisms underlying the maintenance of trophoblast stemness and trophoblast differentiation. Initially, the JEG-3 organoid was used to investigate the central role of YAP in placental stemness (7). As the efficient development of 3D organoid placental models is necessary not only for testing the models but also for expanding the understanding of placentation, the application potential of these models for studying the functions of more specific molecules must be explored (8–10).

Human trophoblasts have unique human-leucocyte-antigen (HLA) expression profiles. HLA-G is a non-classical HLA class I molecule that is exclusively expressed in the EVT and enables “antigen hiding” for choreographed pregnancy events (11). It has limited polymorphism and few encoding splicing possibilities (11). The IMGT/HLA database indicates that HLA-G has eight exons. Exon 1 encodes signal peptides containing coincident fragments for other HLA molecules (12). The remaining exons encode the extracellular α1-, α2-, and α3-domains (exons 2–4), the transmembrane domain (exon 5), and the cytoplasmic domain (exons 6–8). Four membrane-bound (mHLA-G, HLA-G1, -G2, -G3, and -G4) and three soluble (sHLA-G, HLA-G5, -G6, and -G7) protein isoforms are encoded by alternative splicing of the HLA-G gene (12, 13). During pregnancy, HLA-G serves as a tolerogenic ligand that interacts with multiple cell surface receptors, including ILT-2, ILT-4, CD8, CD160, and KIR2DL4 (14). Several studies have characterized the immunomodulatory and other functions of trophoblast HLA-G. However, on the basis of previous experimental methods, such as 2D-culture and siRNA knockdown, the relationship between HLA-G and trophoblast invasion remains controversial (15–17). Furthermore, as an EVT marker gene, the role of HLA-G during trophoblast differentiation is unknown.

Using the published culture protocol from the Nature paper (18), we established the JEG-3 organoid model with cytotrophoblast stemness and validated its ability to differentiate into EVT and explored its ability to form ST. We introduced an HLA-G knockout (KO) JEG-3 cell line using the CRISPR/Cas9 system to investigate the function of HLA-G. Using the HLA-G KO, we also investigated the effect of HLA-G on trophoblast invasion, trophoblast-mediated natural killer cell cytotoxicity, and angiogenesis. Moreover, to investigate the potential roles of HLA-G during trophoblast differentiation, HLA-G knockout JEG-3 cells were first subjected to organoid formation, followed by EVT differentiation. Along with organoid differentiation, exogenous HLA-G protein (presumed sHLA-G) was also administered. Overall, we demonstrated the first application of an organoid model to the functional investigation of HLA-G. Thus, the current study demonstrates the biological and technological potential for HLA-G research advancement in placental development.

## 2 Materials and methods

### 2.1 Cell culture

JEG-3 (HTB-36™, American Type Culture Collection) is an epithelial-like choriocarcinoma cell line with an EVT-like phenotype (KRT7^+^ GATA3^+^ TFAP2C^+^ HLAG^+^) (19). It was cultured in DMEM/F12 supplemented with 10% fetal bovine serum (FBS).

### 2.2 Establishment of the JEG-3 organoids and generation of EVT cells

The formation of trophoblast organoids has been described previously (6, 18). Briefly, 1 × 10^4^ JEG-3 cells (WT or KO) were resuspended in 30 μl of growth-factor-reduced Matrigel (Corning, USA) and then inoculated into 48-well plates with basic trophoblast organoid culture medium in a humidified incubator at 37°C with 5% CO_2_. Small trophoblast organoids were visible after 7 days.

The organoids were transferred into 24-well plates or 35-mm dishes and induced to differentiate into ST or EVT. The ST medium contained forskolin (2μM), whereas the EVT medium was a sequential treatment with EVT medium containing NRG1 (100 ng/ml) for 8 days followed by EVT medium without NRG1 for 3 days. Either the trophoblast organoids culture medium or the ST/EVT differentiation medium was constructed according to the formulas described by Turco et al. and Okae et al.

### 2.3 Generation of HLA-G knockout JEG-3 cells

HLA-G knockout JEG-3 cells were generated using CRISPR/Cas9-based gene editing technology (20). As described previously, small guide RNAs were synthesized by the Shanghai Sheng Gong Company (21) (**Supplemental Table 1**); they were then cloned into the sg-RNA expression vector pSpCas9(BB)-2A-Puro (Addgene, USA). For transfection, a NEPAGENE (Nepa Gene, Japan) electroporator with a poring pulse (voltage, 150 V; pulse length, 7.5 ms) was used. After transfection, JEG-3 cells were selected using puromycin (2 µg/ml). The efficiency of HLA-G knockout was validated by polymerase chain reaction (PCR) and western blot analysis.

### 2.4 DNA extraction and quantification

Genomic DNA was extracted from gene-edited JEG-3 cells using a QIAamp DNA Mini Kit (Qiagen, USA). A nanodrop ND-2000 spectrophotometer was used to determine DNA quality and concentration. The PCR utilized Premix Taq and the following PCR protocol, 94°C for 30 s, 54.3°C for 30 s, 72°C for 1 min 10 s; 35 cycles. The PCR product was run for 30 min at 150 V in 2% agarose. Subsequently, densitometric analysis was performed using a ChemiDoc image analyzer under UV radiation.

### 2.5 RNA sequencing and bioinformatics analysis

At Sangon Biotech (Shanghai) Co., Ltd., HiSeq XTen sequencers were used to perform an RNA sequencing experiment (lllumina, San Diego, CA, USA). After ensuring the integrity of the sequenced data using FastQC (Version 0.11.2), the raw reads were filtered and mapped to the reference genome using HISAT2 (Version 2.0) with the default parameters. The alignment outcomes were analyzed using RAeQC (Version 2.6.1). DESeq2 (Version 1.12.4) was employed to identify differentially expressed genes (DEGs) between the specified groups. Genes were considered as significantly differentially expressed if q-val was <0.05 and |FoldChange| was >2. Subsequently, the functional analysis of DEGs, including Gene Ontology (GO) and Kyoto Encyclopedia of Genes and Genomes (KEGG) enrichment, was performed to determine which DEGs were significantly enriched in specific GO terms or pathways using R (Version 4.2.0) and Rstudio (Version 2022.02.3). GO terms and KEGG pathways with a false discovery rate (q-val) of <0.05 were deemed significant. Transcripts per million (TPM) was used for specific gene set expression, thereby eliminating the influence of gene lengths and sequencing discrepancies and enabling direct comparison of gene expression between two samples.

### 2.6 Reverse transcription-quantitative polymerase chain reaction

Total RNA was extracted using TRIzol reagent (Gibco, USA) in accordance with the manufacturer’s instructions and then reverse transcribed using a Prime-Script RT Reagent Kit (TaKaRa, Japan). Quantitative PCR was conducted using an ABI 7500 Real-Time PCR system (Life Tech, USA) using SYBR Green Reagents (TaKaRa, Japan). In addition, glyceraldehyde 3-phosphate dehydrogenase (GAPDH) was used to normalize the gene expression levels. Subsequently, the relative gene expression levels were calculated using the threshold cycle (CT) method (2−^△△CT^ method). All the primers used for real-time PCR are listed in **Supplementary Table 1**.

### 2.7 Western blotting analysis

Total proteins extracted from JEG-3 cells or JEG-3-ORGs were lysed in lysis buffer containing a protease inhibitor mixture and quantified using Bradford reagent (Solarbio, China). The lysates were separated by SDS-polyacrylamide gel electrophoresis and then transferred to a polyvinylidene fluoride membrane. The membranes were incubated overnight at 4°C with primary antibodies against human HLA-G (1,1,000, Abcam, UK), PAPPA2 (1,1,000, Abcam, UK), and β-tubulin (1,5,000, proteintech, China); they were incubated subsequently with horseradish peroxidase-conjugated rabbit or mouse secondary antibodies (1,10,000, Abcam, UK). After treatment with enhanced chemiluminescence reagent (Meilunbio, China), a ChemiDoc image analyzer was used for densitometric analysis. β-tubulin served as the loading control.

### 2.8 Immunofluorescence

The organoids were washed with cell recovery solution (Corning, USA) before being fixed for 30 min in 4% paraformaldehyde. After permeation and blocking, primary antibodies against human GATA3 (1,100, Abcam, UK), TFA2A (1,100, Abcam, UK), TFA2C (1,100, Abcam, UK), beta-hCG (1,500, Abcam, UK), E-cadherin (1,250, Abcam, UK), HLA-G (1,50, Abcam, UK), and PAPPA2 (1,100, Abcam, UK) were added and incubated at 4°C overnight. The following day, the organoids were sequentially incubated with fluorescence-labeled secondary rabbit or mouse antibody. Phalloidin and DAPI were added accordingly. Subsequently, the stained organoids were imaged using a ZEISS LSM 700 Confocal Laser Scanning Microscope and ZEN imaging software.

### 2.9 Methyl thiazolyl tetrazolium assay

Cell proliferation was measured using cell counting kit-8 reagent (GlpBio, USA) with 2-(2-methoxy-4-nitrophenyl)-3-(4-nitrophenyl)-5-(2, 4-disulfophenyl)-2H-tetrazolium sodium salt (WST-8). Briefly, 1×10^4^ JEG-3 cells in each group were inoculated into a 96-well plate with 100 μl of culture medium per well. After 24 h of cultivation, 10 μl of WST-8 was added to each well and incubated at 37°C for 1 h. A colorimetric analysis was performed at a wavelength of 450 nm.

### 2.10 Matrigel invasion assay

Invasion assays were conducted using Corning BioCoat Matrigel invasion chambers (BD, USA) with 8.0 μm PET membranes in 24-well plates (Corning, USA). Culture medium (600 μl) containing 10% FBS was added to the lower compartment, while 200 μl of serum-free medium containing 1×10^5^ JEG3 cells (HLA-G KO cells or negative control cells) was added to the upper compartment. After culturing for 24 h, the Transwells were fixed with methanol and stained with crystal violet. Finally, the invaded cells were counted under a light microscope.

### 2.11 Angiogenesis assay

Matrigel was polymerized on 24-well culture plates by coating them with undiluted Matrigel (300 μl/well) at 37°C for 30 min. HUVEC cells (5×10^4^) were pre-labeled with Green fluorescence CMFDA for 30 min before being seeded into each well to form endothelial cellular networks. After 4 h, Red fluorescence CMPTX dye-labeled JEG-3 cells (5×10^4^) with or without HLA-G were added to each well and incubated for 24 h. Finally, red and green fluorescence images were captured using a ZEISS Axio Observer fluorescence microscope.

### 2.12 NK92 cytotoxicity assay

Initially, JEG-3 (1×10^5^) cells were labeled with Red fluorescence CMPTX dyes and seeded into a 12-well plate. Subsequently, NK92 cells (1×10^6^) were added to each well. After 24 h of culture, co-culture cells were harvested, blocked with human IgG in RPMI1640 with 1% FBS, and stained with Alexa Fluor^®^ 700-conjugated anti-human CD45 (1,200 BioLegend, US), APC-conjugated anti-human HLA-G (1,200 BioLegend, US), and 7-AAD staining solution (1,400, BioLegend, USA) for 30 min. Subsequently, the data were collected using a Deflex B5-R3-V5 Flow Cytometer (405/488/638) and analyzed using the FlowJo_v10.6.2 software. All the antibodies used for this study are described in **Supplementary Table 2**.

### 2.13 Exogenous treatment of recombinant HLA-G protein in EVT generation

In conjunction with EVT differentiation from JEG-3 organoids (HLA-G KO group), the sequential EVT medium was supplemented with recombinant HLA-G protein (OriGene, USA) at varying concentrations (0.025 µg/ml, 0.05 µg/ml, and 0.1 µg/ml) as described previously (22). On days 0, 3, 6, and 9, the EVT-differentiated JEG-3 organoids (HLA-G KO group) were harvested for RNA analysis.

### 2.14 Statistical analysis

SPSS Statistics version 25.0 (IBM) was used for the statistical analysis. Using GraphPad Prism 8.0, the data were graphically represented as mean ± standard deviations (SD). In addition, the independent t-test and one-way analysis of variance were used to determine the differences between two and multiple groups, respectively. Each experiment was conducted a minimum of three times. *P <*0.05 was regarded as the statistical significance threshold.

## 3 Results

### 3.1 Establishment and characterization of trophoblast organoids from JEG-3 cells

**Figure 1:**
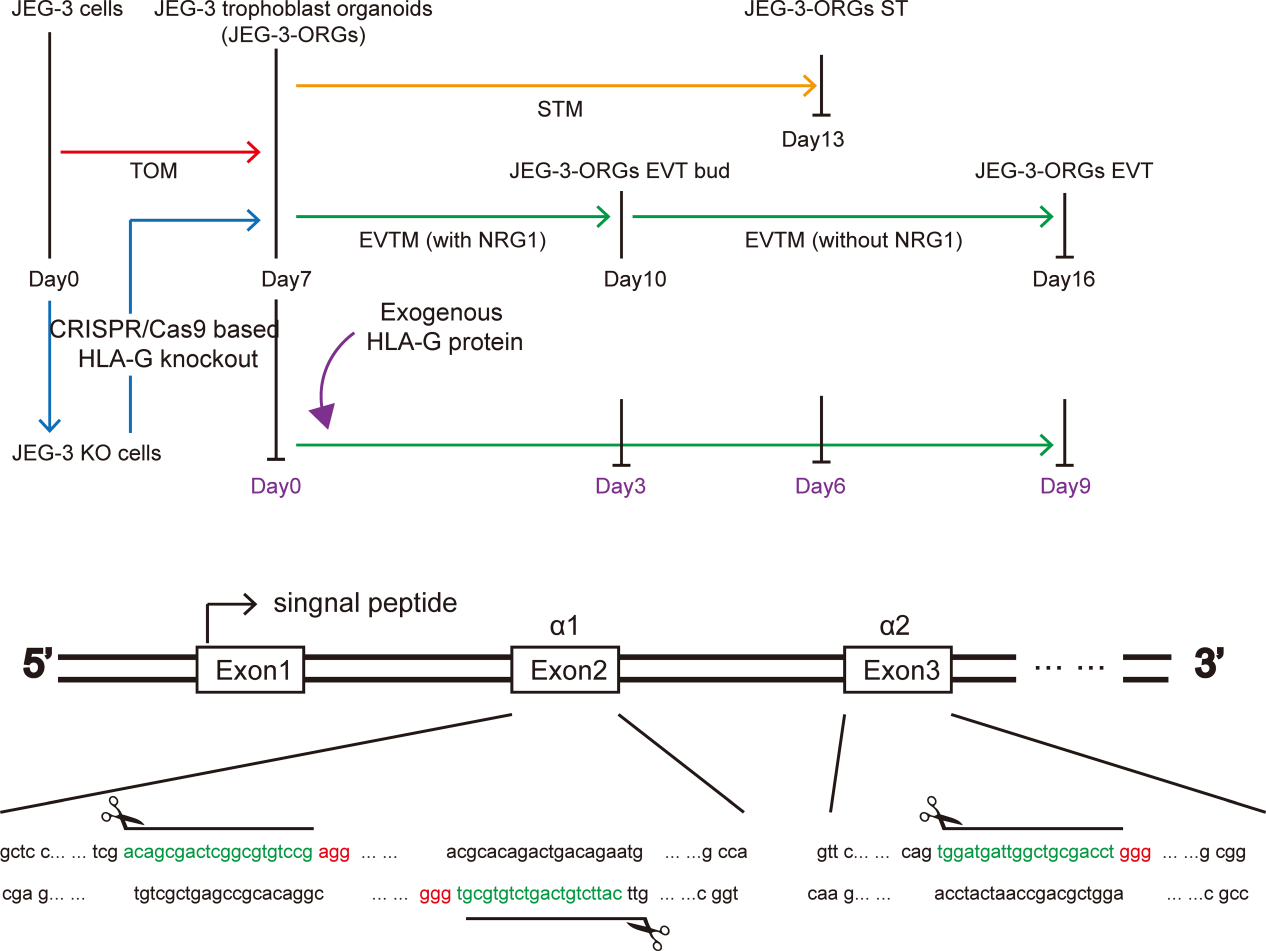
HLA-G study based on JEG-3 trophoblast organoids and the CRISPR/Cas9 approach. **(A)** Schematic illustration of the formation of the trophoblast organoid and differentiation model. Red, orange, green, blue, and purple represent TB-ORG formation, ST differentiation, EVT differentiation, HLA-G knockout, and exogenous HLA-G treatment, respectively. **(B)** Genomic sequence for the HLA-G knockout targeted region. The PAM sequences are indicated in red. The small guide RNA sequences are underlined in green.

**Figure 2:**
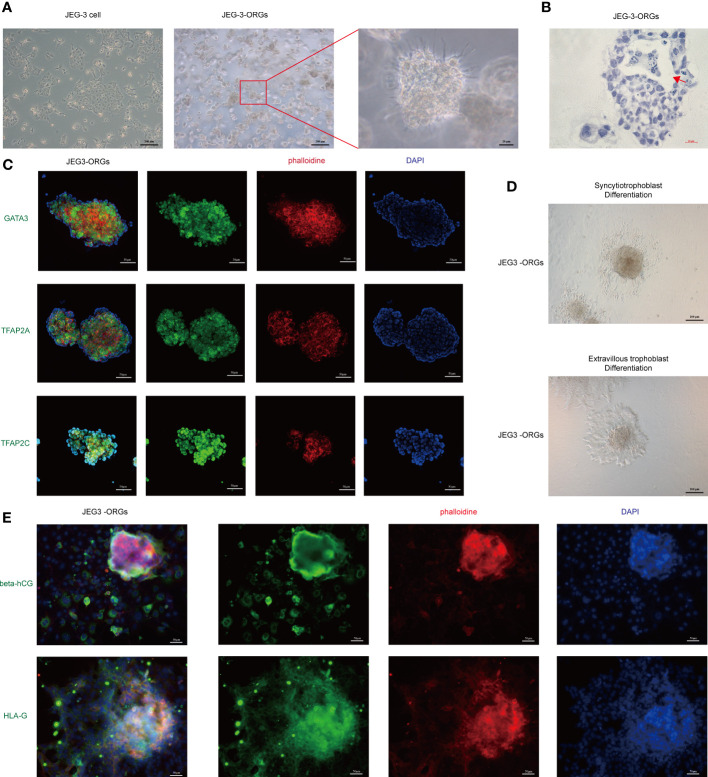
Formation of trophoblast organoids through the reprogramming of JEG-3 cells. **(A)** Images of the different cultured statuses of JEG-3 cells. Left, two-dimensional culture; middle and right, three-dimensional culture in the middle and right. **(B)** Images of hematoxylin staining of the cross-section of JEG-3-ORGs. The red arrow indicates the intercellular vacuoles. **(C)** Immunofluorescence staining images of GATA3, TFAP2A, and TFAP2C in JEG-3-ORGs. **(D)** The generation of syncytiotrophoblast and extravillous trophoblast from JEG-3-ORGs. **(E)** Immunofluorescence staining images of beta-hCG and HLA-G at the ST and EVT differentiation stages of JEG-3-ORGs, respectively.

The cytotrophoblast organoid medium containing activators (EGF, HGF, FGF2, CHIR99021, and R-spondin-1) and inhibitors (A83-01 and Y-27632) targeting the Wnt, MAPK, cAMP/AKT, TGF-beta, and ROCK signaling pathways was applied to the formation of JEG-3 organoids. JEG-3 cells were successfully reprogrammed into trophoblast organoids (JEG-3-ORGs) following a 7-day culture (**Figures 1A**, **2A**). It has intercellular vacuoles and a three-dimensional growth pattern (**Figure 2B**). JEG-3-ORGs expressed trophoblast markers GATA3, TFAP2A, and TFAP2C, just like organoids derived from the human primary trophoblast (**Figure 2C**). To examine the potential for the differentiation of JEG-3-ORGs, organoids were directed towards ST and EVT. During ST/EVT differentiation, the presence of beta-hCG/HLA-G-positive cells at the periphery of JEG-3-ORGs indicated the ST-like or EVT-like nature of the cells (**Figures 2D, E**).

**Figure 3:**
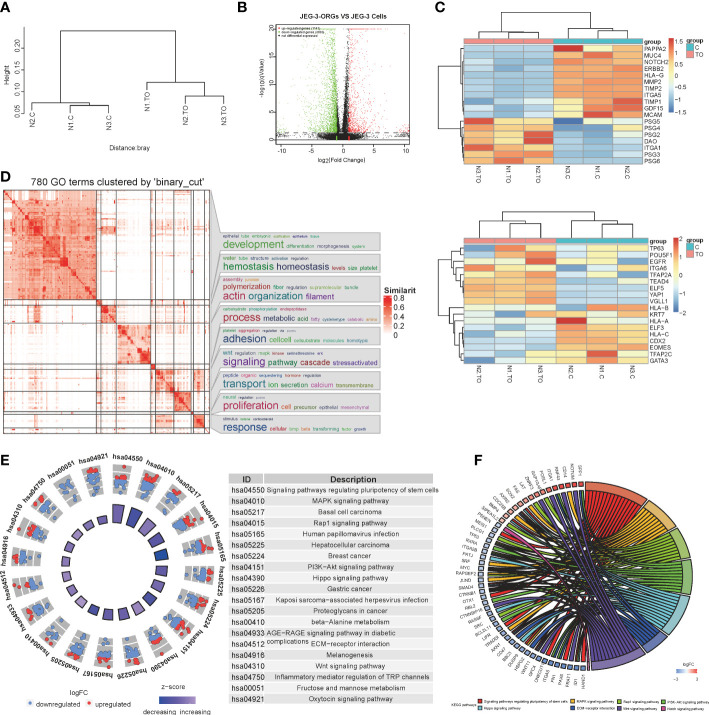
RNA-sequencing analysis for the process of JEG-3-ORG formation. **(A)** Hierarchical clustering for JEG-3-ORGs and JEG-3 cells. **(B)** Volcano plot showing DEGs of the JEG-3 cell reprogramming. Red points represent significantly upregulated genes, green points represent significantly downregulated genes, and gray points represent genes that were not significantly differentially expressed. The criteria for DEGs is a |log2FC|>2 and a q-value of <0.05. **(C)** Clustered heat map showing expression of the selective identity markers for subtype trophoblasts. The upper panel shows ST- and EVT-classified genes and the lower panel shows TSC and CTB markers. **(D)** Simplified GO terms enrichment. **(E)** Circle diagram of the top 20 KEGG enriched signaling pathways. **(F)** Chord plot for crosstalk, covering the top 7 enriched canonical pathways.

To further characterize the identity of JEG-3-ORGs, a comparison of their transcriptome profiles with those of JEG-3 cells was performed. JEG-3-ORGs and JEG-3 cells formed two clusters *via* hierarchical clustering (**Figure 3A**), indicating their distinct molecular identities. Using differentially expressed gene (DEG) analysis, 1,141 upregulated and 3,093 downregulated genes were identified in the JEG-3-ORGs relative to the parental JEG-3 cells (**Figure 3B**). JEG-3-ORGs were in a cytotrophoblast-like state, as indicated by a heatmap of marker gene expression. With the exception of DAO and ITGA1, the majority of EVT markers (such as MMP2, ITGA5, PAPPA2, and ERBB2) were downregulated. Except for *GDF15*, genes belonging to the pregnancy-specific glycoprotein family (PSG) were upregulated as ST markers (**Figure 3C** upper). Nevertheless, few TSC and CTB markers were altered (e.g., *TP63*, *EGFR*, *POU5F1*, *ITGA6*, *KRT7*, and *TFAP2C*). Only TE-related genes (e.g., *CDX2* and *EOMES*) were downregulated (**Figure 3C** below). *HLA-G* and *HLA-C* were downregulated in anticipation. *ELF5* was highly expressed, which was consistent with its reported hypomethylated status, whereas *ELF3* was weakly expressed, indicating that it was hypermethylated. This change in marker genes indicates that the EVT-like fate of JEG-3 cells in TO-ORGs medium has been predominantly reversed to a CTB-like state. On the basis of the transcriptomic data, we also investigated the strongly involved pathways regulating the organoid formation in JEG-3 cells and found that the DEGs during reprogramming were enriched in GO terms converging on development and metabolic processes (**Figure 3D**), which were consistent with those reported in the primary trophoblast ORGs (5). The top 20 KEGG-enriched signaling pathways included the “MAPK”, “PI3K-Akt”, and “Wnt” pathways (**Figure 3E**). These three pathways also cover most of the activators and inhibitors for the trophoblast organoid medium. In addition, the crosstalk for the top eight enriched development-relevant pathways, represented by a chord diagram, showed that the JEG-3-ORGs were in the germinative stage, with downregulated bHLH transcription factors (such as HAND1, ID1, and MYC) and TGF-beta signaling (e.g., SMAD4) (**Figure 3F**). Altogether, JEG-3-ORGs can be produced in the published trophoblast organoid medium and, to a certain extent, have similar properties to human primary TB-ORGs for further trophoblast functional study.

### 3.2 CRISPR/Cas9-based HLA-G knockout-mediated receptor-dependent function in JEG-3 cells

**Figure 4:**
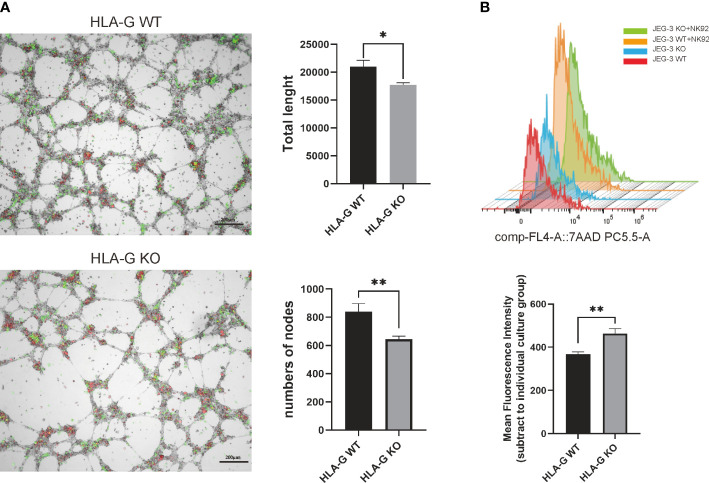
HLA-G functional assay on the JEG-3 cells co-cultured model. **(A)** Images and representative statistical bar graphs of an angiogenesis assay in Jeg-3 cells (green) transfected with HLA-G knockout or empty vectors under a 24 h co-culture with HUVEC cells (red). **(B)** Colored histogram (left) and mean fluorescence intensity (right) of 7AAD staining (PC5.5-A) results with cell viability between JEG-3 KO and WT cells in co-culture with NK92 cells. *P<0.05 or **P<0.01. n = 3 in triplicate.

To generate a JEG-3 cell line devoid of HLA-G, the CRISPR/Cas9 system was used. As the extracellular domain α1 encoded by exon 2 is the common peptide for seven HLA-G protein isoforms (G1 to G7), two small guide RNAs (sg-RNAs) targeting exon 2 and one sg-RNA targeting exon 3 (**Figure 1B**) were designed and used in conjunction to ensure the deletion of nearly all known HLA-G isoforms. After electroporation and drug selection, a successful HLA-G knockout JEG-3 cell line (JEG-3 KO cells) was established, and its efficacy was first validated by PCR and western blotting (**Figure S1A**). Additional functional validation was performed on the well-established functions of HLA-G, including the inhibition of natural killer cell cytotoxicity and the promotion of endothelial cell angiogenesis. When co-cultured with HUVEC, JEG-3 KO cells exhibited impaired tube remodeling (**Figure 4A**); when co-cultured with NK92, JEG-3 KO cells exhibited increased cytotoxicity compared with wild-type JEG-3 cells (**Figure 4B**). Overall, the results demonstrated the successful generation of an HLA-G JEG-3 knockout cell line using CRISPR/Cas9, which could be used for future functional studies of HLA-G.

In addition to examining the effect of HLA-G on proliferation and invasion ability in JEG-3 KO cells using wild-type JEG-3 as a control, we examined the effect of HLA-G on proliferation in JEG-3 KO cells. The CCK8 proliferation assay revealed no significant difference between the JEG-3 cells of the wild-type (WT) and KO genotypes (**Figure S1B**). Likewise, the Transwell invasion assay revealed no significant difference between the KO and WT cells (**Figure S1C**). The mRNA expression of invasion-related genes (*MMP2*, *MMP9*, *TIMP1*, and *TIMP2*) was also unaffected by the absence of HLA-G (**Figure S1D**). To compare the global molecular profile, we sequenced the transcriptomes of both JEG-3 KO and wild-type cells. Analysis of differential gene expression revealed only a few significant genes, indicating that HLA-G knockout has a minimal effect on the functions of JEG-3 cells (**Figure S1E**).

In this section, we used the CRISPR/Cas9 system to generate a validated molecular and functional HLA-G knockout JEG-3 cell line. Transcriptome and functional assay analysis of the HLA-G KO model demonstrated that HLA-G knockout had a minimal effect on the proliferation and invasion of JEG-3 cells. However, when co-cultured with NK92 and HUVEC, the effect of HLA-G knockout on cytotoxicity and angiogenesis was significant, indicating that HLA-G is a molecule that has an effect that is dependent on its coupling receptors, which may in turn indirectly affect JEG-3 cells.

### 3.3 HLA-G knockout minimally affected JEG-3-ORG formation

**Figure 5:**
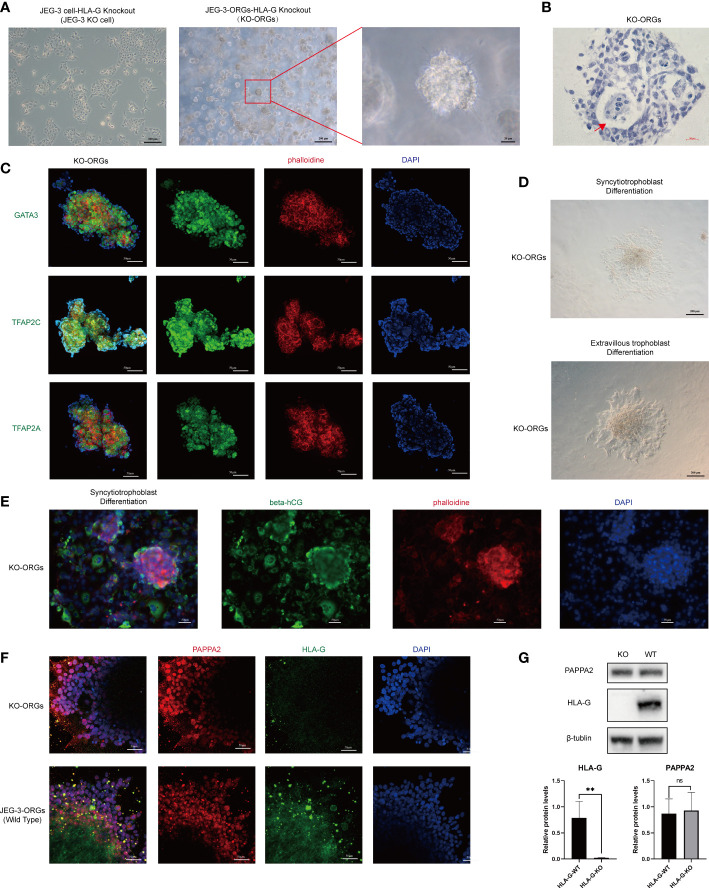
Formation of trophoblast organoids through reprogrammed JEG-3 KO cells. **(A)** Images of the different cultured statuses of JEG-3 KO cells. Left, two-dimensional culture; middle and right, three-dimensional culture. **(B)** Hematoxylin staining images of the cross-section of KO-ORGs. The red arrow indicates the intercellular vacuoles. **(C)** Immunofluorescence staining images of GATA3, TFAP2A, and TFAP2C in KO-ORGs. **(D)** Generation of syncytiotrophoblast and extravillous trophoblast from KO-ORGs. **(E)** Immunofluorescence staining images of beta-hCG at the ST differentiation stage of KO-ORGs. **(F)** Immunofluorescence staining images of PAPPA2 and HLA-G at the EVT differentiation stage of two kinds of JEG-3-ORGs. **(G)** Protein expression of HLA-G and PAPPA2 at the EVT differentiation stage of two kinds of KO-ORGs. Top, blot; bottom, quantification of relative protein levels. **P<0.01; ns, not significant. n = 3 in triplicate.

With JEG-3 KO cells, we attempted to generate trophoblast organoids in the same manner as JEG-3 WT cells (**Figure 1A**). Interestingly, the JEG-3 KO cells-derived ORGs (KO-ORGs) exhibited similar morphology to WT-ORGs (**Figures 5A, B**). The KO-ORGs also expressed GATA3, TFAP2A, and TFAP2C (**Figure 5C**) and had the ability to differentiate into ST cells, as indicated by beta-hCG (**Figures 5D** upper, **5E**). Additionally, upon directed EVT differentiation, migration was observed in KO-ORGs (**Figure 5D** below). As HLA-G, the commonly used EVT marker, was the investigated target that was knocked out in our study, PAPPA2, another traditional EVT marker, was used instead to confirm the differentiation of EVTs from organoids (**Figure 5F**). When comparing differentiated EVT cells from KO-ORGs and WT-ORGs, there was no significant difference in PAPPA2 protein expression (**Figure 5G**).

The formation of JEG-3-ORGs without HLA-G was further characterized by comparing the transcriptomic profiles of JEG-3 KO cells and their organoids. Two distinct clusters were revealed by hierarchical clustering (**Figure S2A**). Between these two groups, a total of 3,675 DEGs (1,227 upregulated genes and 2,448 downregulated genes) were identified (**Figure S2B**). Except for *DAO*, *PSG2*, *PSG6*, and *PAPPA2*, the heatmap of ST- and EVT-characteristic genes exhibited similar changes to the formation of organoids from JEG-3 WT cells (**Figure S2C** upper). With the exception of EGFR, GATA3, and TFAPAPA, clusters of TSC and CT markers, which were mentioned previously in WT-ORGs, also exhibited similar changes (**Figure S2C** below). Nonetheless, an integrated GO term analysis revealed that the formation of KO-ORGs could be approximately summarized as the biological process of development and signal pathway response (**Figure S2D**), indicating that JEG-3 KO cells were activated by stress during reprogramming. The top 20 KEGG-enriched pathways for the formation of KO-ORGs are also involved in the “MAPK” and “PI3K-Akt” signaling pathways, except for the “Wnt” pathway (**Figure S2E**). Additionally, several regulatory components of the mutual signaling pathway were somewhat distinct in KO-ORGs (**Figure S2F**). In MAPK signal, FASLG, rather than FAS, TP53 and SRF, is regulated in KO-ORGs. In addition, JAG1 and JAG2, not the PSENEN, were regulated in KO-ORGs in response to the Notch signal. Overall, the findings demonstrated that JEG-3-ORGs could be generated from HLA-G knockout JEG-3 cells in the identical culture medium.

### 3.4 HLA-G treads different reprogram paths that lead to the same JEG-3-ORGs destination

**Figure 6:**
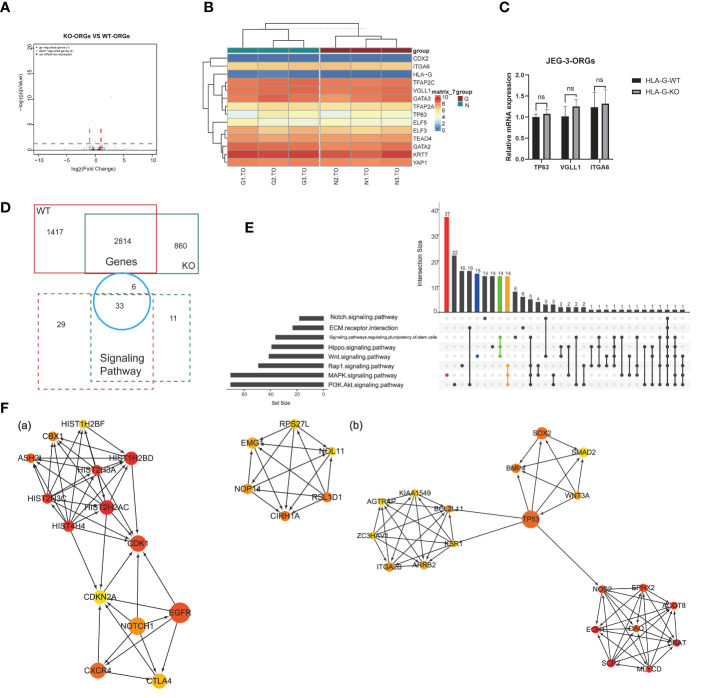
Comparison analysis for different DEGs in JEG-3-ORG formation. **(A)** Volcano plot showing DEGs between KO-ORGs and WT-ORGs. **(B)** Clustered heat map showing the expression of the selective TSC and CTB classified genes. **(C)** Quantification of relative mRNA expression of ITGA6, VGLL1, and TP63 between KO-ORGs and WT-ORGs. **(D)** Diagram of the shared or individual genes and signaling pathways for two types of JEG-3 cell reprogramming. **(E)** UpSet plot showing intersections among the common signaling pathways. Coloured bars show genera/taxa exclusively observed in the “MAPK”, “PI3K/Akt” and “Wnt” signaling pathways and intersective pathways. **(F)** PPI network analysis of the individual DEGs expressed in KO-ORGs and WT-ORGs. Ten hub genes were selected using the plug-in cytoHubba of Cytoscape based on MCC scores. **(A)** Network based on 1,417 individual DEGs for the WT group. **(B)** Network based on 859 individual DEGs for the HLA-G KO group. ns, not significant.

Comparison of the KO-ORGs and WT-ORGs *via* transcriptome sequencing revealed no significant difference. The similarity was demonstrated by a volcano plot (**Figure 6A**). The heatmap for the TSC- and CT-classified gene clusters revealed that their undifferentiated status was comparable (**Figure 6B**). The similar expression of mRNA for TP63, VGLL1, and ITGA6 further supported their identity (**Figure 6C**).

Although similar, a comparison of the different DEGs during reprogramming in the presence and absence of HLA-G could be instructive. The 2,184 DEGs shared between KO-ORGs and WT-ORGs enriched 33 KEGG pathways, which overlapped with the individual analyses (**Figure 6D**). In accordance with the regulatory components of the culture medium, the “MAPK” and “PI3K-Akt” signaling pathways were the dominant developmentally significant pathways, irrespective of the HLA-G (**Figure 6E**). Interestingly, in our JEG-3ORGs, the Rap1 signaling pathway ranked first. In addition, it comprises one of the planar cell polarity-related pathways. To trace how the trophoblast was still able to develop in the absence of HLA-G, a PPI network was constructed using the individual DEGs [1,417 genes (WT group) and 859 genes (KO group)] (**Figure 6F**). The relationship of the top 20 hub gene clusters between the absence of HLA-G (e.g., HIST2H2AC, CDK1, CXCR4, and CIRH1A) and the presence of HLA-G (e.g., CRAT, DAO, TP53, BMP4, and BCL2L11) may be a trigger for compensation mechanisms of HLA-G-relative trophoblast development. Even though DEGs were unequal between HLA-G KO and WT, HLA-G was not required for the formation of JEG-3 -ORGs.

### 3.5 EVT generation from JEG-3 organoids is unaffected by HLA-G

**Figure 7:**
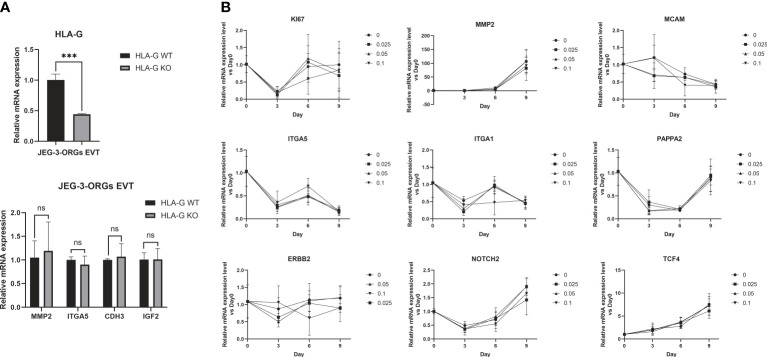
Generation of EVT from JEG-3-ORGs with the effect of HLA-G. **(A)** Quantification of the relative mRNA expression of HLA-G, MMP2, ITGA5, CDH3, and IGF2 at the EVT differentiation stage of two kinds of JEG-3-ORGs (KO-ORGs and WT-ORGs). **(B)** Temporal expression of the relative mRNA quantification for the acknowledged EVT-classified genes after exogenous addition of HLA-G protein-based KO-ORGs at different concentrations (0.025 µg/ml, 0.05 µg/ml, and 0.1 µg/ml). ***P<0.001; ns, not significant. n = 3 in triplicate.

HLA-G is gradually expressed in EVT during early pregnancy and gradually decreases as the pregnancy progresses (13). This leads us to the question of whether HLA-G facilitates the generation of EVTs. Under the cytotoxicity of Cas9 protein and the stress of electrotransfection, the compact structure of trophoblast organoids renders them fragile. Therefore, genetic modification of all TB-ORGs remains a difficult task. Instead, HLA-G knockout TB-ORGs were derived directly from JEG-3 cells that are susceptible to CRISPR-Cas9-mediated editing. First, we directly compared the differentiation process between WT-ORGs and KO-ORGs on the basis of their previously attested nearly equivalent identities (**Figure 1A**). The mRNA expression of conventional EVT identification markers (MMP2, ITGA5, CDH3, and IGF2) following EVT differentiation exhibited no significant changes, indicating that HLA-G knockout had no effect on EVT production (**Figure 7A**). In addition to the knockout, recombinant HLA-G protein was overexpressed through exogenous addition (**Figure 1A**). During differentiation between the KO and HLA-G addition groups, the mRNA expression of EVT-recognized markers (MMP2, ITGA5, ITGA1, ERBB2, MCAM, NOTCH2, PAPPA2, and TCF4) did not change significantly at any concentration (0.025 µg/mL, 0.05 µg/mL, and 0.1 µg/mL) (**Figure 7B**). Briefly, HLA-G played an inconsequential role in EVT differentiation based on JEG-3-ORGs.

## 4 Discussion

During pregnancy, the placenta is an essential organ for the absorption of nutrients, the exchange of metabolic waste, and the synthesis of hormones (23). The appearance of trophoblast organoids that mimic physiological states can facilitate the mechanistic investigation of placental development. HLA-G is uniquely expressed in the EVT and is necessary for a healthy pregnancy (24–26). CRISPR/Cas9-based gene-editing technology is a robust method for manipulating gene expression (20). Correspondingly, the study of trophoblast HLA-G function can be advanced by integrating organoid and CRISPR/Cas9 techniques. In this study, we generated an HLA-G knockout JEG-3 cell line and JEG-3-ORGs to investigate HLA-G function in the trophoblast. However, we observed receptor-dependent effects of HLA-G on trophoblast function and differentiation.

Even though JEG-3 cells originate from choriocarcinomas with a genetic signature distinct from that of a normal trophoblast, it is the only highly expressed HLA-G trophoblast cell line and the only line worthy of use in HLA-G-related research. Accordingly, we successfully created a JEG-3 trophoblast organoid resembling cytotrophoblast with the robust capacity to differentiate into EVTs. For ST differentiation through forskolin and EGF treatment, which successfully induced multinucleated syncytium in the trophoblast stem cell differentiation system (data not shown), only beta-hCG-expressing cells without observable multinucleated syncytium were generated in our JEG-3-ORG system. The results are consistent with reported findings that the formation of syncytium from JEG-3 cells requires additional conditions (27) (28). Therefore, the application potential of the JEG-3-ORG system for studying ST formation requires further investigation. When selecting a culture model for placental development research, its ability to replicate the *in vivo* microenvironment is crucial. In our study, we used a culture medium for the formation of primary human trophoblast organoids, which resulted in organoids that replicated the *in vivo* maternal-fetal environment (5). Using the organoid model, extravillous trophoblast developmental events could be investigated extensively. By examining the formation of JEG-3 organoids from the more committed EVT-like JEG-3 cells, some hints about trophoblast differentiation were uncovered. In Turco’s medium, the focus of culture supplementation has been the activation or inhibition of signaling pathways associated with proliferative cells. Early in the first trimester, Wnt, TGF-beta, and MAKP signaling pathways are active in trophoblast progenitors (29–31). In addition to the known signaling pathways, our bioinformatics analysis revealed that during the organoid formation process (reprogramming), the signaling pathways “regulating pluripotency of stem cells”, Rap1, Hippo, “ECM-receptor interaction”, and Notch changed dramatically in the JEG-3 cell with the characteristics of a mature EVT (32, 33). The regulation of these signaling pathways may provide hints for improving trophoblast organoid models and investigating the mechanism of EVT differentiation. According to a comprehensive characterization analysis, the generated JEG-3-ORGs resembled the primary TB-ORGs not only in their organoid identity but also during the reprogramming process, which resulted in the enrichment of the two most prominent GO terms, development and metabolic process (5). Although JEG-3 cells cannot fully represent primary trophoblasts, their derived JEG-3-ORGs might aid researchers in identifying the pathological mechanisms underlying early developmental defects in relevant pregnancies.

To generate HLA-G knockout JEG-3-ORGs, CRISPR/Cas9-based HLA-G knockout was performed on JEG-3 cells, which were then reprogrammed into KO-ORGs. The SgRNAs used in this study were proposed in previous publications, ensuring total HLA-G knockout. The studies only examined the effect of knockout on NK cell cytotoxicity (21, 34). It appears that the interaction between HLA-G and its receptor KIR2DL4, which is expressed on NK cells, could reduce the cytotoxicity of NK cells. In addition, our study was consistent with these HLA-G receptor-dependent results. Using the HLA-G KO model as a starting point, our study investigated the effects on additional trophoblast functions. The relationship between invasiveness and EVT-expressed HLA-G is one of the branches of placental development mechanisms. Previous studies have reported that HLA-G5 treatment increases the invasiveness of primary trophoblast and JEG-3 *via* KIR2DL4/LILRB1/ERK signaling (22). By contrast, the action was not observed in the trophoblast cell line SGHPL-4 (17). In addition, HLA-G1 had a negligible effect on the invasion of trophoblast cell lines JAR and HTR8 under hypoxic conditions when HGF was induced (16). In our study, the absence of HLA-G in JEG-3 cells had no significant effect on cell invasion, which is consistent with a number of previously published studies (16, 17). Future application of CRISPR/Cas9 technology to primary trophoblast will be required to reach a consensus on the role of HLA-G in trophoblast invasiveness. The same holds true for trophoblast proliferation, which this study found to be minimally affected by HLA-G knockout.

Surprisingly, KO-ORGs and WT-ORGs exhibited striking similarities in morphology and trophoblast marker gene expression. If HLA-G is considered as the sole determinant of trophoblast lineage, these findings suggest that the signaling pathways involved in Turco’s medium facilitate the reprogramming of all subtypes of trophoblasts. Despite this, something intriguing was discovered during comparative reprogramming. For example, DAO was upregulated during the formation of JEG-3-ORGs in the presence of HLA-G but was unaffected during the HLA-G KO-related reprogramming process. DAO, as a result of histamine intolerance, is believed to prevent the excessive entry of bioactive histamine into the maternal or fetal circulation, as well as inhibit the prolonged exposure of decidual cells (35). It is detectable in a subset of EVTs near veins and arteries and is diminished in early onset preeclampsia (36). Consequently, HLA-G may serve as the link between DAO and EVT. Further investigation of the distinct DEGs involved in the formation of KO-ORGs or WT-ORGs can contribute to mechanism research for trophoblast subpopulations during placental development. Moreover, the expression of the histone family during reprogramming in the absence of HLA-G indicates that epigenetic modification may have occurred. As healthy individuals possess HLA-G null alleles, such as G*0105N, which do not encode functional HLA-G1 or HLA-G5 (37), other hub genes (e.g., *CDK1*, *ASH2L*, *CXCR4*, *RSL1D1*, and *CTLA4*) associated with the cell cycle and ribosome biogenesis may be candidates for compensating for the absence of HLA-G. By contrast, other hub genes clustering with the presence of HLA-G (*SCP2*, *NOS2*, *DAO*, *TP53*, *SOX2*, and *KSR1*) may be the regulator for HLA-G-associated disease monitoring.

Primary EVTs with restricted physiological HLA-G expression are susceptible and lose their natural phenotype gradually after 3–4 days of *in vitro* culture (38). To date, no reliable medium for the long-term cultivation of EVTs exists. The Wnt signaling pathway is currently recognized as regulating EVT differentiation (39). This EVT differentiation medium, developed by Turco et al., contains TGF-beta inhibitors but no growth factors or Wnt activators. NRG1 is only added during the initial few days (3, 40). Interestingly, on the foundation of extremely similar KO-ORGs and WT-ORGs, EVTs were normally developed without HLA-G, as well as with the addition of HLA-G protein. In other words, HLA-G is not necessary for the differentiation of EVT based on JEG-3-ORGs. However, further research is required to determine whether HLA-G interacts with the Wnt signaling pathway in EVT differentiation. Considering that EVTs have also expressed other HLA molecules, such as HLA-C, -E, and -F, attention should be paid to the interaction between them. The above trophoblastic HLA molecules are mentioned in a recent study of placental villous tissues (41). HLA-G and -C were more highly expressed in PE, whereas HLA-E and -F were reduced. However, HLA-G functional research in EVTs with other placental HLAs would be more informative.

In order to investigate the characteristics of HLA-G in trophoblast development, we combined trophoblast organoid culture technology with the CRISPR/Cas9 gene-knockout system and bioinformatics. Using our JEG-3-ORG model, we discovered that the absence of HLA-G had minimal effects on organoid formation and trophoblast differentiation. Although comparable EVTs could be generated from JEG-3-ORGs without HLA-G, it is still unknown whether the function of terminal EVTs are affected by HLA-G. Accordingly, this topic requires additional research. HLA-G appears to be a molecule that functions in the presence of its receptors, based on the negative effects of HLA-G knockout in JEG-3 cells on the function of co-cultured NK and HUVEC cells. To determine whether the negative effects of HLA-G knockout on cells in the microenvironment have a feedback effect on trophoblast function/differentiation, additional research is required. Thus, to contribute to HLA-G studies, additional advanced research on trophoblast culture medium could be conducted.

In summary, JEG-3-ORG was shown to be a useful model for studying extravillous trophoblast differentiation due to the easier gene-editing property of the JEG-3 cell line compared with primary cells. As a model, JEG-3-ORGs demonstrated advantages in the study of trophoblast differentiation and could be used as an embryo surrogate for the investigation of interactive activities with the endometrium, which cannot be realized in two-dimensional JEG-3 culture. However, both models can be complementary to each other for the research of different trophoblast functions (**Table 1**). Even though, in this study, the JEG-3-ORG model revealed that HLA-G plays a limited role in EVT differentiation, it is still advantageous to use TB-ORGs as a rapid model for trophoblast research, such as in drug screening, EVT generation, or other molecule studies. From a perspective view, there are also limitations in this study. First, the recombinant HLA-G protein is susceptible to functioning as soluble isoforms. To determine whether the membrane-bound HLA-G antigens act similarly, additional research is required. Moreover, owing to the lack of multinucleated STs from JEG-3-ORGs with the current ST differentiation protocol, the application potential of JEG-3-ORGs for studying ST differentiation is limited. Lastly, the signaling pathways and EVT markers they covered, as well as the imperfect cavities present in the JEG-3-ORGs, indicated that this model may partially represent the real events of *in vivo* placental structure. Therefore, the generalization of our findings should be cautioned. To investigate placentation events in the future, the use of primary villous tissue combined with gene-editing technology is highly recommended, with the expectation of producing different results.

**Table 1:** Comparison between two-dimensional JEG-3 and JEG-3-ORGs.

		2D JEG-3	JEG-3-ORGs
**Difference**	Culture medium	10% FBS+DMEM/F12	Turco’s medium (5), B. Dietrich’s medium (6)
	Morphology	Monolayered epithelial cell, EVT-like phenotype (19)	Multilayered epithelial spheroids, Stem cell-like phenotype (6)
	Functional research	Invasion (16, 17), NK cytotoxicity (42), Angiopoiesis	Trophoblast differentiation, Interaction with endometrium
**Same**		Choriocarcinoma-derived, expressed HLA-G, drug screening	

## Data availability statement

The data presented in the study are deposited in the NCBI sequence read archive (SRA) repository, accession number: PRJNA942198.

## Author contributions

Y-QY and WY conceived of the presented idea and revise and polish the manuscripts. B-MZ, D-DC and X-FL carried out the experiment and wrote the manuscript with support from PC and C-LL. X-LL and LW contributed to the sample collection. Y-GDn helped do data collection. All authors contributed to the article and approved the submitted version.

## Glossary

**Table:**

HLA-G	Human leucocyte antigen G
EVT	extravillous trophoblasts
WT	wild type
KO	knockout
TB-ORGs	trophoblast organoid
CTBs	cytotrophoblasts
STB	syncytiotrophoblast
MHC	major histocompatibility complex
sg-RNAs	small guide RNAs
DEGs	differentially expressed genes;
TPM	transcripts per million
GO	Gene Ontology
KEGG	Kyoto Encyclopedia of Genes and Genomes
TE	trophectoderm
TSC	trophoblast stem-cell
TESC	trophectoderm stem cell
CCTs	cell column trophoblasts
Wnt	Wingless-Type
TGF-beta	transforming growth factor beta
MAPK	Mitogen-Activated Protein Kinase;
PI3K-Akt	Phosphatidylinositol-3-kinase protein kinase B;
GATA3	GATA Binding Protein 3
TFAP2A	Transcription Factor AP-2 Alpha
TFAP2C	Transcription Factor AP-2 Gamma;
CDX2	Caudal Type Homeobox 2
TP63	Tumor Protein P63;
EGFR	Epidermal Growth Factor Receptor
POU5F1 (OCT4)	POU Class 5 Homeobox 1
KRT7	Keratin 7
ELF5	E74 like ETS transcription factor 5
HAND1	Heart and neural crest derivatives expressed 1
ID1	inhibitor of DNA binding 1
MMP	Matrix Metallopeptidase
TIMP	Tissue Inhibitor Of Metalloproteinase;
ITGA	Integrin Subunit Alpha
ERBB	Erb-B2 Receptor Tyrosine Kinase
DAO	diamine oxidase
PAPPA2	Pappalysin 2
IGF	insulin-like growth factor
GDF	Growth Differentiation Factor;
PSG	pregnancy specific glycoprotein family
HIST	histone
SAMD4	SMAD Family Member 4
CTNNBIP1	Catenin Beta Interacting Protein 1
MCAM	Melanoma Cell Adhesion Molecule
TCF4	Transcription Factor 4
SOX2	SRY-Box Transcription Factor 2.
