# Supplementary material for: Application of a JEG-3 organoid model to study HLA-G function in the trophoblast
Source: Front Immunol. 2023 Mar 15;14:1130308. doi: 10.3389/fimmu.2023.1130308 (PMC10050466; doi:10.3389/fimmu.2023.1130308)
Supplement: Supplementary file 1 [file DataSheet_1.zip › Figure S1.DOCX]

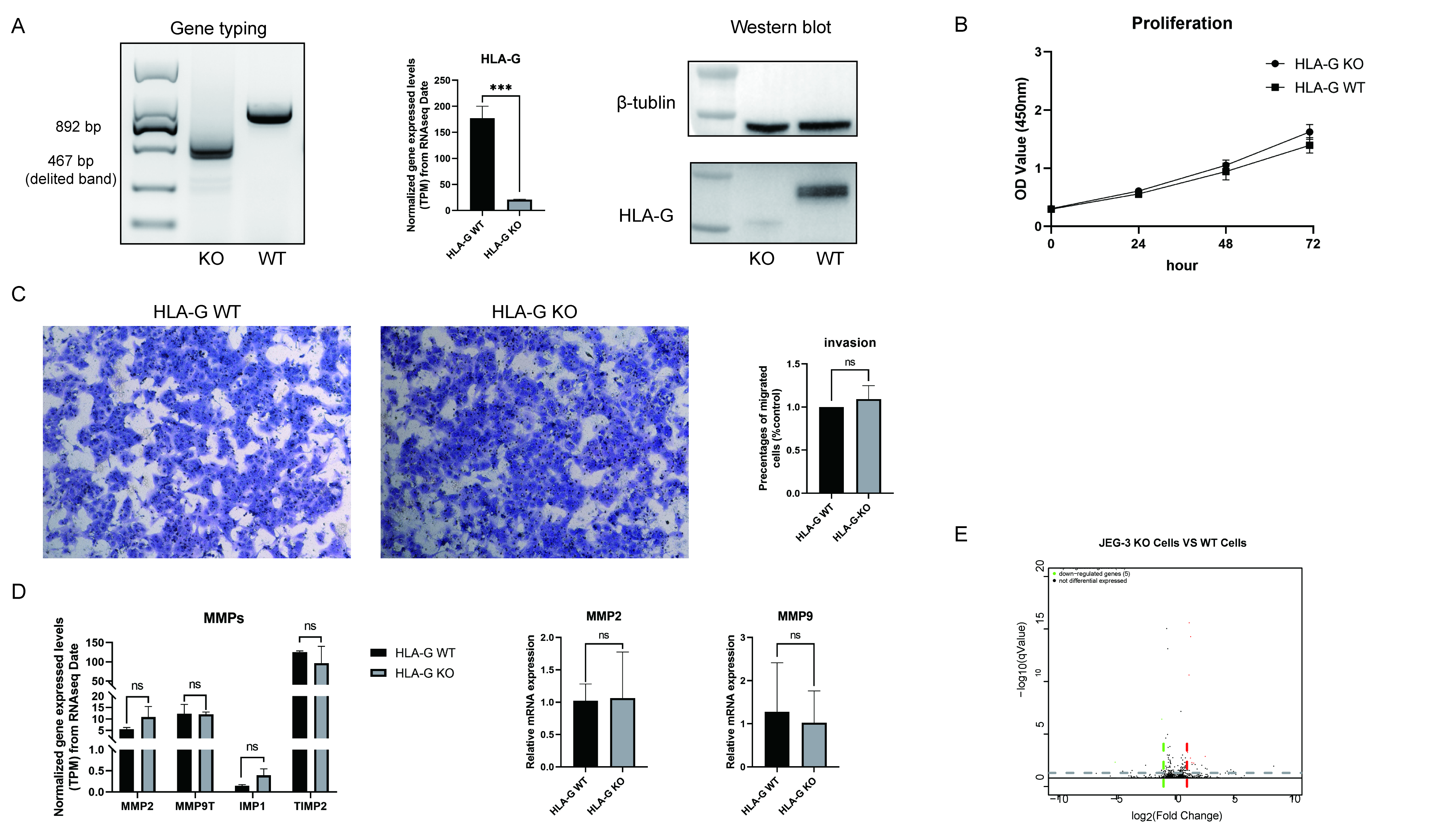


Figure S1. Establishment of JEG-3 KO cell utilized CRISPR/Cas9 based HLA-G knockout technology. A. The verification of HLA-G knockout efficiency on JEG-3 cells. Genomic DNA expression (Left), TPM expression (Middle) and Protein expression (Right). B. Proliferation of JEG-3 KO and WT cells in CCK8 assay. C. Images and representative statistical bar graphs of matrigel invasion assay in Jeg-3 cells transfected HLA-G knockout or empty vector. D. TPM data of MMPs and quantification of relative mRNA levels of MMP2 and MMP9 for JEG-3 KO and WT cells. E. Volcano plots showing no DEGs between JEG-3 KO and WT cells. *P*** <0.01 or *P**** <0.001 means a significant difference. *P* = ns means no statistic difference. n = 3 in triplicate.
