# Supplementary material for: Application of a JEG-3 organoid model to study HLA-G function in the trophoblast
Source: Front Immunol. 2023 Mar 15;14:1130308. doi: 10.3389/fimmu.2023.1130308 (PMC10050466; doi:10.3389/fimmu.2023.1130308)
Supplement: Supplementary file 1 [file DataSheet_1.zip › Figure S2.DOCX]

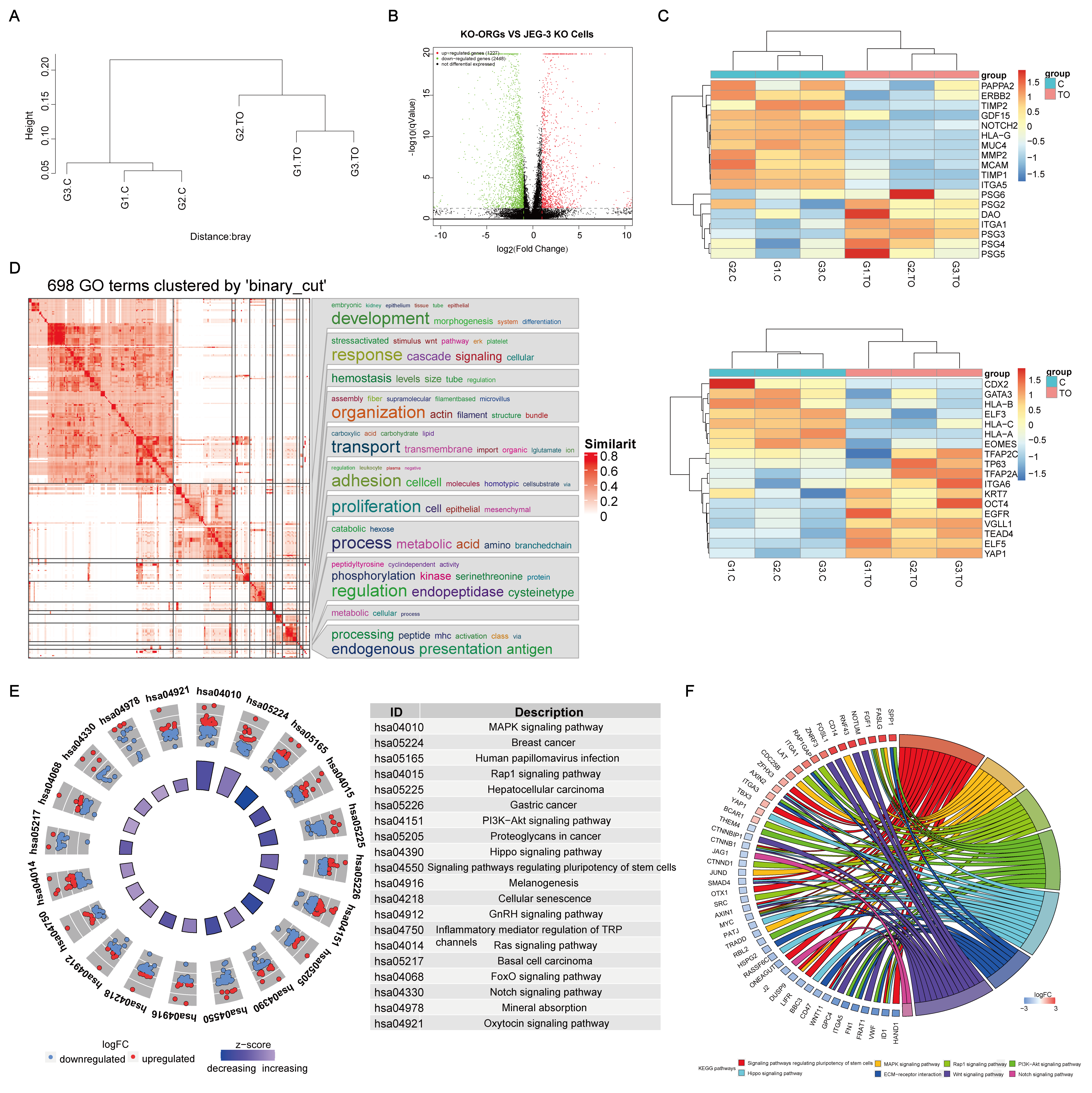


Figure.S2 RNA-sequencing analysis for the process of KO-ORGs formation. A. Hierarchical clustering for KO-ORGs and JEG-3 KO cells. B. Volcano plots showing DEGs of the JEG-3 KO cell reprogramming. C. Clustered heat map showing expression of the selective identity-markers for subtype trophoblasts. Upper is ST- and EVT- classified genes. Below is TSC and CTB markers. D. Simplify GO terms enrichment. E. Circle Diagram for top20 KEGG enriched signaling pathways. F. Chord plot for crosstalk covered the top7 enriched canonical pathway.
