# Supplementary material for: Application of a JEG-3 organoid model to study HLA-G function in the trophoblast
Source: Front Immunol. 2023 Mar 15;14:1130308. doi: 10.3389/fimmu.2023.1130308 (PMC10050466; doi:10.3389/fimmu.2023.1130308)
Supplement: Supplementary file 1 [file DataSheet_1.zip › Table S1.DOCX]

Supplementary Table1. Nucleotide base sequences designed in ShangHai ShengGong company.

| 1.1 CRISP/Cas9 based sgRNA coding oligos: | | |
| --- | --- | --- |
| sgRNA1: 5′-CACCGACAGCGACTCGGCGTGTCCG-3′;  sgRNA2: 5′-CACCGGGTCGCAGCCAATCATCCAC-3′;  sgRNA3: 5′-CACCGTCATTCTGTCAGTCTGTGCG-3′. | | |
| 1.2 RT-PCR Primers: | | |
| Gene | FORWARD (5’>3) | REVERSE (5’>3) |
| HLA-G | GTGGCTCCACAGATACCTG | CGTCCTGGGTCTGGTCCT |
| MkI67 | CAGACATCAGGAGAGACTACAC | GTTAGACTTGCTGCTGAGTCTA |
| MMP2 | CAGTACCGAGAGAAAGCCTATT | CAGGATGTCATAGGTCACGTAG |
| MMP9 | CAGTACCGAGAGAAAGCCTATT | CAGGATGTCATAGGTCACGTAG |
| ITGA5 | CATGATGAGTTTGGCCGATTTG | CCCCCAGGAAATACAAACACTA |
| CDH3 | GAATCAAAAGATCAGCTACCGC | AATCAGTGTTAGCAGAAGGGTT |
| ITGA1 | AGCTCCGCGTCTACAAAGC | AGCTCCGCGTCTACAAAGC |
| ERBB2 | TGCAGGGAAACCTGGAACTC | ACAGGGGTGGTATTGTTCAGC |
| MCAM | AGCTCCGCGTCTACAAAGC | AGCTCCGCGTCTACAAAGC |
| NOTCH2 | TGAGAACATTGATGATTGTGCC | GTGGGCAGGTGCAAATATATTG |
| PAPPA2 | GAAAGCCACCATCTTGATTAGC | CCAGACTGGTCTAGACTGCTAG |
| TCF4 | CAAGCACTGCCGACTACAATA | CCAGGCTGATTCATCCCACTG |
| ITGA6 | GAGCATGATGAAAGTCTCGTTC | ACTTCATGTCTCTCTTCAGCAA |
| VGLL1 | CCAAAGGCAAACAGAAGCCTA | CATCACACCTTCACTCTGACTC |
| TP63 | AGGACACGTCGAAACTGTGC | GGACCAGCAGATTCAGAACGG |
| GAPDH | GGAGCGAGATCCCTCCAAAAT | GGCTGTTGTCATACTTCTCATGG |
