# Supplementary material for: Application of a JEG-3 organoid model to study HLA-G function in the trophoblast
Source: Front Immunol. 2023 Mar 15;14:1130308. doi: 10.3389/fimmu.2023.1130308 (PMC10050466; doi:10.3389/fimmu.2023.1130308)
Supplement: Supplementary file 1 [file DataSheet_1.zip › Table S2.DOCX]

Supplementary Table 2. Antibodies list.

| Name | manufacturer | Cat.no |
| --- | --- | --- |
| PC5.5-conjugated anti- human 7AAD | BioLegend | 420403 |
| AF700-conjugated anti-human CD45 | BioLegend | 368513 |
| APC-conjugated anti-human HLA-G | BioLegend | 335909 |
| Anti HLA-G monoclonal antibody[MEM-G/1] | Abcam | ab7759 |
| Anti HLA-G monoclonal antibody[MEM-G/9] | Abcam | ab7758 |
| Recombinant Mouse IgG1 monoclonal Isotype control | Abcam | ab280974 |
| Anti betaHCG monoclonal antibody | Invitrogen | MA514680 |
| Recombinant Rabbit IgG monoclonal Isotype control | Abcam | Ab125938 |
| Anti GATA3 monoclonal antibody | Abcam | ab282110 |
| Anti TFAP2A monoclonal antibody | Abcam | ab108311 |
| Anti TFAP2C monoclonal antibody | Abcam | ab218107 |
| Anti PAPPA2 ployclonal antibody | Novus | NBP1-76487 |
| Anti β-tublin polyclonal antibody | proteintech | 10068-1-AP |
| Goat Anti-Mouse IgG HRP antibody | Abcam | ab205719 |
| Goat Anti-Rabbit IgG HRP antibody | Abcam | Ab205718 |
